# Treatment of rats with spinal cord injury using human bone marrow-derived stromal cells prepared by negative selection

**DOI:** 10.1186/s12929-020-00629-y

**Published:** 2020-02-18

**Authors:** Lorenzo Romero-Ramírez, Siyu Wu, Johannes de Munter, Erik Ch. Wolters, Boris W. Kramer, Jörg Mey

**Affiliations:** 1 Hospital Nacional de Parapléjicos, c/Finca la Peraleda, 45071 Toledo, Spain; 2 School of Mental Health and Neuroscience and EURON Graduate School of Neuroscience, Maastricht University, Universiteitssingel 40, 6229ER Maastricht, Netherlands; 3 Neuroplast BV, Urmonderbaan 22, 6167RD Geleen, Netherlands

**Keywords:** Spinal cord injury, Stem cells, Rat, Inflammation, Human, Bone marrow

## Abstract

**Background:**

Spinal cord injury (SCI) is a highly debilitating pathology without curative treatment. One of the most promising disease modifying strategies consists in the implantation of stem cells to reduce inflammation and promote neural regeneration. In the present study we tested a new human bone marrow-derived stromal cell preparation (bmSC) as a therapy of SCI.

**Methods:**

Spinal cord contusion injury was induced in adult male rats at thoracic level T9/T10 using the *Infinite Horizon impactor*. One hour after lesion the animals were treated with a sub-occipital injection of human bmSC into the *cisterna magna*. No immune suppression was used. One dose of bmSC consisted, on average, of 2.3 million non-manipulated cells in 100 μL suspension, which was processed out of fresh human bone marrow from the iliac crest of healthy volunteers. Treatment efficacy was compared with intraperitoneal injections of methylprednisolone (MP) and saline. The recovery of motor functions was assessed during a surveillance period of nine weeks. Adverse events as well as general health, weight and urodynamic functions were monitored daily. After this time, the animals were perfused, and the spinal cord tissue was investigated histologically.

**Results:**

Rats treated with bmSC did not reject the human implants and showed no sign of sickness behavior or neuropathic pain. Compared to MP treatment, animals displayed better recovery of their SCI-induced motor deficits. There were no significant differences in the recovery of bladder control between groups. Histological analysis at ten weeks after SCI revealed no differences in tissue sparing and astrogliosis, however, bmSC treatment was accompanied with reduced axonal degeneration in the dorsal ascending fiber tracts, lower Iba1-immunoreactivity (IR) close to the lesion site and reduced apoptosis in the ventral grey matter. Neuroinflammation, as evidenced by CD68-IR, was significantly reduced in the MP-treated group.

**Conclusions:**

Human bmSC that were prepared by negative selection without expansion in culture have neuroprotective properties after SCI. Given the effect size on motor function, implantation in the acute phase was not sufficient to induce spinal cord repair. Due to their immune modulatory properties, allogeneic implants of bmSC can be used in combinatorial therapies of SCI.

## Background

In spinal cord injury (SCI) cellular degeneration and the disruption of connections between the brain and the body cause paralysis and the loss of sensory and autonomic functions. Worldwide, the incidence of SCI ranges from 13 to 163 per million people per year, depending on the country [1]. Over two thirds are due to trauma (falls, traffic and sport-related accidents, gun shots) and the rest to non-traumatic SCI (spinal stenosis, tumors, vascular ischemia). In addition to the devastating loss of quality of life to the patients, SCI causes a large economic burden to their families and society. Although rehabilitation therapy has continuously improved since the 1950s, there is no curative treatment of SCI [1, 2].

Spinal cord injury triggers local and systemic secondary mechanisms resulting in a chronic inflammatory state, which is mainly responsible for extensive cell death [3]. These mechanisms are addressed with one available pharmacological treatment, namely the application of a high dose of methylprednisolone (MP) within the first hours after the injury. Following three independent clinical trials in the 1980s and 1990s (National Acute SCI Studies [4]) MP became a standard intervention [5]. However, subsequent clinical experience showed that it is often ineffective and causes severe side effects such as higher incidence of sepsis, gastrointestinal haemorrhage or pulmonary embolism [6]. Thus, new therapies of SCI are highly desired.

A promising strategy to cure neurodegenerative pathologies is based on the application of stem cells [7–9]. In the first studies these were intended to replace lost neurons or glia [10], and with neural stem cells this continues to be an objective [11, 12]. With non-neuronal stem cells, on the other hand, the main rationale consists in modulating the inflammatory response [7, 13]. Paracrine factors and extracellular vesicles that are released from mesenchymal stem cells are expected to prevent secondary degeneration and to support a regenerative remodeling after SCI [8, 14, 15]. An easily accessible source of this type of cells is the bone marrow, which contains hematopoietic and mesenchymal stem cells. Different from induced pluripotent stem cells [16], bone marrow-derived stromal cells (bmSC) pose no risk of tumor formation. We have developed a novel procedure to prepare fresh human bmSC with low immunogenicity (Neuroplast BV, patent WO2015/059300A1). This preparation is based on the depletion of erythrocytes and lymphocytes from bone marrow extracts without substantial manipulation or cultivation of the isolated cells.

The objective of the present study was to assess the safety and therapeutic benefits of acute intrathecal injection of the novel bmSC preparation in SCI-lesioned rats and to compare it with acute intraperitoneal injection of MP. So far, one other study has been published with these bmSC as a treatment of SCI [13]. In these experiments, cell suspensions were injected into the spinal cord of immune compromised rats after a balloon compression injury. This treatment was associated with an improvement in the recovery of motor function at two and five weeks after SCI but not at one, three and four weeks. While serum levels of IL-1β and TNFα were reduced, treatment had no consistent effect on neuroinflammation in the spinal cord. Based on the outcome, the present investigation has implemented the following design: 1) We have tested the bmSC in immune competent animals. Previously, T-cell deficient rats were used in order to avoid a possible immunological rejection to the human implants. Since one expected mechanism of action consists in the suppression of inflammation, the use of immune competent rats was considered necessary. 2) As suggested by the EMA, stem cell treatment was compared with MP. Despite its limited efficacy, MP is still the only FDA-approved pharmacological treatment of SCI [4, 5], and any new intervention should therefore be compared with this standard. 3) A different SCI model was chosen. While the balloon compression used before is a validated model to investigate physiological consequences of SCI, a much larger number of human SCI cases (about half of all [8]) are represented by the contusion injury model. 4) Rats were monitored up to 9 weeks after SCI to assess the long term effects of implanted bmSC which may be observed in the chronic stage. 5) Additional outcome measures in the present study included tests of motor function (Rotarod), autonomic function (bladder control), neuropathic pain (tactile allodynia) and a more extensive histological assessment including axonal degeneration and macrophage activation.

## Methods

### Experimental animals

**Fig. 1:**
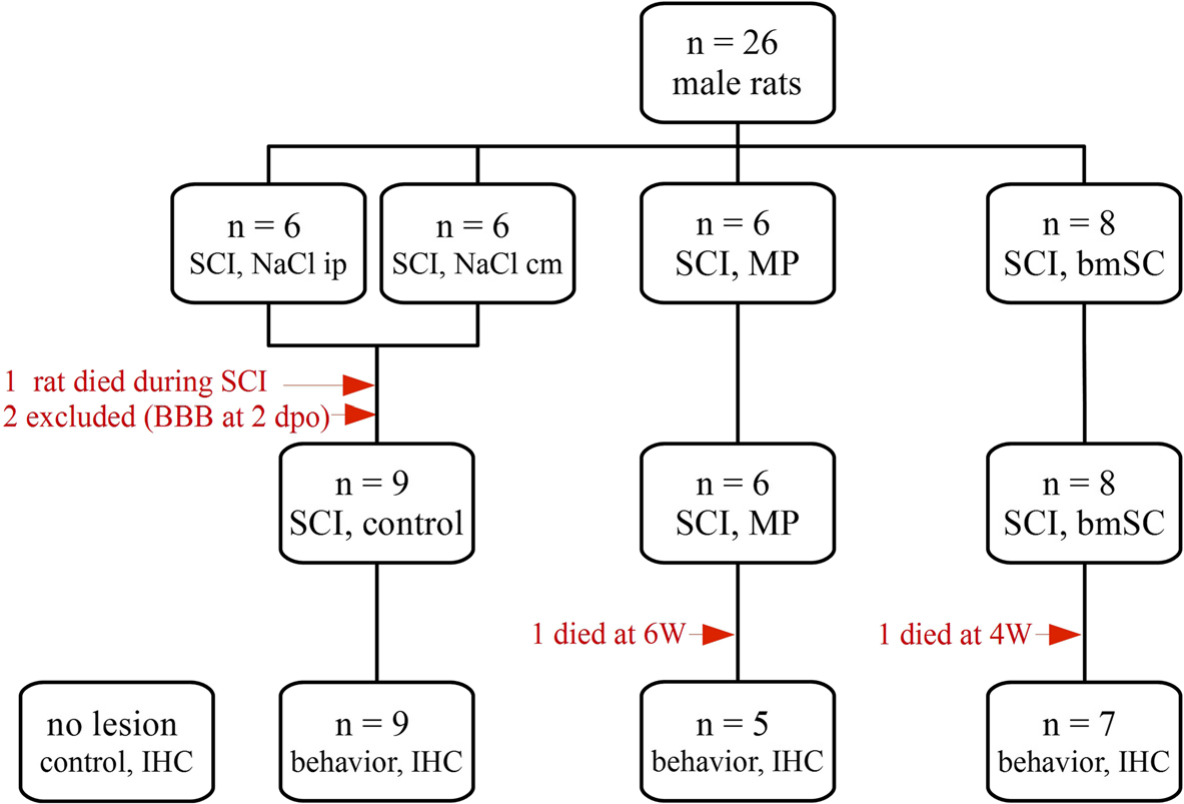
Experimental plan and treatment groups. Animals that received spinal cord contusion injury were pseudo-randomly assigned to four groups. Rats with intraperitoneal and intrathecal (cisterna magna) saline injections were planned to be evaluated as one control group unless behavioral evaluation showed statistical differences between them. One animal was lost due to bleeding during spinal cord surgery, two animals had to be excluded from the study because open field evaluation revealed an incomplete lesion (BBB at 2 dpo) and two rats died during the observation period. In the histological evaluation, treatment groups were also compared to tissue samples of non-injured animals

The ethics committee for Animal Care of the *Hospital Nacional de Parapléjicos* reviewed experimental protocol, surgical procedures and post-operational care (163CEEA/2017), which were subsequently approved by the *Consejería de Agricultura y Ganadería de Castilla-la Mancha* (ref. 210,498, following EU directive 2010/63/EU). During the acclimatization period, six to eight weeks old male Wistar rats (*Rattus norwegicus*; mean weight 304 g +/− 14 g, raised in the animal facility of the hospital), were kept in pairs under standard housing conditions (12 h light/dark cycle, humidity 40–60%, temperature 22 °C) with ad libitum access to food and water. Following surgery, animals were kept in separate cages. A total of 26 animals entered the study (Fig. 1). In an exploratory experiment to determine the least invasive way of implanting the stem cells (intrathecal injection in the spinals cord vs. cisterna magna), additional 10 rats had been operated and their motor recovery was followed over a period of 3 weeks.

### Spinal cord contusion injury

To induce anesthesia the rats were exposed to 5% isoflurane/95% oxygen in a plexiglass chamber. During surgery, anesthetic was reduced to 2.5% isoflurane/oxygen breathed via an inhalation mask of a vaporizer (flow rate 0.4 L/min; Medical Supplies and Services). Fifteen minutes before surgery, rats were weighed and received the analgesic buprenorfine 0.05 mg/kg (Buprex 0.03 mg/mL). After induction of anesthesia, the fur on the back was shaved. The animals were then taped to the operating table, their body temperature being maintained on a heat pad with a rectal thermometer. Corneal dehydration was prevented with ophthalmic ointment (Lubrithal).

Surgery was performed with the help of an operating microscope (Leica). After skin incision and dissection of the muscle layers covering the vertebrae, serrate muscles were spread with a retractor (Reda 19,621–07), and the spinal cord was exposed by laminectomy of vertebrae T9-T11 without damaging the dura mater (small rongeur FST 16021–14, scalpel with round blade, two round forceps mid size, one forceps with teeth, sterilized cotton-tips, gelatin sponge). To suspend the spinal cord for applying the contusion injury, the dorsal spinal processes T7 and T12 were fixed with the clamps of the impactor device (Infinite Horizon, IH). The impactor rod was positioned centrally at T9/T10 over the spinal cord midline, and the contusion was applied by pressing it against the dorsal surface of the tissue. When a force of 2 N was reached, the rod was immediately retracted (zero dwell time). The device was calibrated before every experiment. We checked the procedure visually (hematoma) and by monitoring the IH displacement/time and force/time plots. In two cases, where the impactor rod hit a bone, the laminectomy was extended and the contusion injury repeated.

Following this procedure, the rats were released from the IH clamps. The wound was covered with subcutaneous fat tissue from the same animal. Overlying muscles were re-apposed and sutured, and the skin was closed with a non-interrupted intradermal suture (resorbable thread 4.0) and disinfected with iodine. Animals were then disconnected from anesthesia and received 2 × 2.5 mL isotonic saline s.c. and antibiotic treatment marbofloxacine 5 mg/kg (Marbocyl 10 mg/mL, s.c.).

### Postoperative treatment and care

Following surgery, rats were housed individually to prevent biting at skin sutures. Throughout the study, we performed daily overall health assessments, including inspection of the animals’ well-being, body weight, urodynamic assessments and routine checks to detect urinary tract infections. For the first 3 days animals received two daily s.c. injections of buprenorfine 0.05 mg/kg for pain relief. Subcutaneous injections of 5 mg/kg marbofloxacine were given at the day of surgery, at 2 and 4 days post operation (dpo). Postsurgical care also included food pellets soaked in water and a water bottle with longer tube. The bladders were checked twice daily and voided manually until the rats were urinating spontaneously. The volume of retained urine was recorded. In case of urinary infection, the animals were treated with marbofloxacine 5 mg/kg s.c. every 48 h until the urine was clear and without blood. Euthanasia at the end of the study was induced by i.p. injection of 100 mg/kg of sodium pentobarbital (Dolethal).

### Experimental groups

Animals were pseudo-randomly assigned to four experimental groups, which all received the same SCI but differed in the treatment procedure (Fig. 1). Group 1 received five NaCl i.p. injections, the first immediately after SCI and subsequently every 8 h; group 2 received one 100 μL NaCl injection into the cisterna magna at 1–2 h after SCI; group 3 was treated with five MP injections i.p., one after SCI and subsequently every 8 h; group 4 was treated with one 100 μL bmSC injection into the cisterna magna at 1–2 h after SCI. Rats were assigned random identifiers, which were written with permanent marker on their tails. During the following 9 weeks of behavioral evaluation, care takers and investigators were blinded regarding the experimental condition of the individual animals. To keep the number of experimental animals low, the control groups (NaCl i.p. and per cisterna magna) were planned to be joined in one statistical group unless significant differences were found in the behavioral tests.

### Preparation of bmSC

Bone marrow-derived cells for SCI treatment were prepared at the Neuroplast facility, Geleen, Netherlands, under GMP conditions. The cells were not expanded by cultivation (Neurocells, patent WO2015/059300A1). Recruitment of volunteers for bone marrow collection, procedures and documentation were approved by the ethics committee of Maastricht University Medical Center (METC 13–2-032). From two donors (BM31, BM33) 50 mL bone marrow was collected. Clotting was prevented by EDTA adjuvants. The fresh bone marrow was immediately processed, using automated Ficoll density gradient centrifugation to remove the erythrocytes and reactive proteins. Subsequently, B-cells (CD20+), T-cells (CD3+), monocytes (CD14+) and natural killer cells (CD56+) were removed using antibody-based cell sorting with magnetic beads (negative selection; CliniMacs Plus, Miltenyi Biotec GmbH). The viability and cell type composition of each batch was analyzed with flow cytometry (CD34, CD271, CD90, CD105, CD73). For the present study, cells were cryoprotected with DMSO, frozen in liquid nitrogen, shipped on dry ice to Toledo, Spain, and then stored in liquid nitrogen until use. Cell viability was again determined after thawing, i.e. immediately before application in vivo (cytometry, propidium iodide exclusion). On average, cisterna magna injections contained 2.3 +/− 0.5 × 10^6^ viable cells.

### Intrathecal infusion of bmSC, injections of MP or vehicle

For cisterna magna injections of bmSC or saline, 1.5 h after SCI, animals were re-anesthetized with ketamine 50 mg/kg (Ketolar 50 mg/mL. i.p.) combined with xylacine 5 mg/kg (Sedaxylan 20 mg/mL, i.p.) and one i.p. injection of atropine 0.04 mg/kg. The head and neck of anesthetized rats were shaved, and the animals were positioned in a stereotactic frame (Kopf) with the neck flexed to 70° at the atlanto-occipital joint. Ophthalmic ointment was applied, and the skin was superficially disinfected with 70% ethanol.

Simultaneously, the bmSC were prepared for injection: For the treatment of two rats, one batch containing 1 mL of frozen cell suspension was thawed in a 37 °C water bath, spun down, washed with saline, centrifuged and resuspended in 210 μL saline. From this, 10 μL was removed for cytometric counting of cell numbers and determination of cell viability. The remaining 200 μL cell suspension was kept on ice until the rats were ready for receiving the injections. The setup for slow injection into the cisterna magna consisted of an electric syringe pump and a sterile 1 mL plastic syringe connected to a Fogarty arterial embolectomy catheter 0.67 mm, fixed to the stereotactic device. A steel canula 23G 0.6 mm was used to penetrate the atlanto-occipital membrane before inserting the catheter.

When the anesthetized rat was in place, the atlanto-occipital membrane was accessed by midline anterior-posterior incisions of skin and muscles, which were separated and fixed laterally. Syringe and catheter, previously flushed with sterile saline, were loaded with cell suspension (bmSC treatment) or saline (vehicle treatment) and placed in the holder of the microliter pump. Under microscopic control, the membrane was then punctured and access to the cisterna magna confirmed by observing the appearance of clear cerebrospinal fluid. The catheter was inserted and its content slowly infused (100 μL/3 min) before retracting the catheter. Finally, the muscle and skin were sutured, wiped with Betadine, and the animal was placed in its cage, receiving post-operative care as described for SCI.

Rats belonging to the MP group received in total five i.p. injections of 30 mg/kg MP, given at 1 h after SCI and subsequently one every 8 h. Lyophilized MP was reconstituted to 20 mg/mL just prior to injection and kept at 4 °C for the remaining applications. Rats belonging to the second control group were treated with the same volume of saline, 150 μL/100 g, injected i.p. at the same times.

### Evaluation of locomotor functions in the open field

Recovery of limb movements was evaluated using the Basso/Beattie/Bresnahan (BBB) locomotor function test [17] for 5 min/rat in an open field. The BBB scale ranges from 0 (no hind limb movement) to 21 (normal movements, coordinated gait with parallel paw placement). Scores from 0 to 7 indicate the return of isolated movements in the three joints (hip, knee and ankle). Scores from 8 to 13 indicate the return of paw placement and coordinated movements with the forelimbs. Scores from 14 to 21 show the return of toe clearance during stepping, predominant paw position, trunk stability and tail position. Motor scores were measured pre-SCI (baseline), at 2 dpo, 4 dpo, and once per week for the next 9 weeks after lesioning. At the beginning, we established a criterion of BBB < 2 at 2dpo for inclusion in the study because a higher score was considered to indicate incomplete SCI. Scoring was performed by two independent investigators who were blinded with respect to the treatment of the individual animal. Following independent assessment, both investigators discussed their reasons and independently awarded their score.

### Rotarod locomotor function test

The Rotarod test [18], which required the rats to maintain their body on a rotating rod, was performed according to the instruction manual of the manufacturer (Ugo Basile SRL, Gemonio, Italy). In four training sessions of 5 min each, which were administered two and 1 days before SCI surgery, all rats learned this task at a constant speed of 5 rpm of the rotating rod. Since balancing on the rotating bar cannot be performed by a rat with completely paralyzed hind legs, the first testing was performed at 4 dpo, subsequently at 7dpo and then once per week. In the test runs, the rotation speed was accelerated from 5 rpm to 15 rpm over a period of 3 min. Readout in this assay was the time that the rats were able to stay on the rotating rod before falling off (mean of two repetitions, separated by a break of ≥15 min). Data obtained from rats that refused to hold on to the bar were included in the evaluation because we lacked an independent criterion to distinguish between voluntary refusal and inability to perform the task.

### Von Frey test of mechanical allodynia/hyperalgesia

Before SCI and at the end of the 9 week observation period, tactile allodynia/hyperalgesia was tested manually using a kit of von Frey filaments with a range of different diameters. For this, rats were placed individually in small cages with a wire mesh bottom. To deliver a constant force, a filament with specific diameter was pressed perpendicularly to the plantar surface of the hind paw until it buckled and held for 2–5 s. A response was considered positive when the animal exhibited any nocifensive behavior such as brisk withdrawal or licking of the paw [19]. Both hind paws were stimulated from below, and the paw withdrawal threshold determined using the simplified up-down method [20].

### Tissue preparation and histological staining

Ten weeks after SCI the rats were sacrificed with an overdose of sodium pentobarbital followed by transcardial perfusion with phosphate buffered saline (PBS) and 4% paraformaldehyde/PBS. Spinal cords were prepared, post-fixed for 1 h, then transferred to PBS and stored at 4 °C. For histological processing, 18 mm long spinal cord segments that included the lesion site were dissected, dehydrated, embedded in paraffin and cut in 3 μm transverse sections using a Leica RM2265 microtome. Sections separated by 250 μm were mounted on polylysine-coated glass slides (Superfrost Plus) and stored at 4 °C. To assess the extension of the lesion, the complete series of spinal cord sections of all rats were rehydrated, stained with hematoxylin/eosin (H&E), dehydrated again and cover slipped with Histomount (Merck).

### Immunohistochemistry

Prior to immunohistochemical staining, rehydrated sections were incubated for 30 min at 90 °C (water bath) in 10 mM Na citrate/0.05% Tween 20, pH 6.0, for antigen retrieval. Standard procedure included blocking 1 h at RT with 5% normal goat serum/0.05% Tween 20 in Tris-buffered saline (TBS-T), incubation with primary antibodies for 12 h at 4 °C in a humidified chamber and 2 h incubation with fluorescence-labeled secondary antibodies at RT. Nuclei were stained with 10 μg/mL Hoechst-33342 for 15 min at RT. Sections were cover slipped with Mowiol/DAPCO or ImmuMount (Thermoscientific). We used the following primary antibodies, usually in a double staining protocol in the dilutions indicated in parenthesis:

Rabbit anti-GFAP, polyclonal (Sigma G9269; 1/500), rabbit anti-caspase-3/activated (Calbiochem PC679; 1/200), rat anti-MBP, polyclonal (Abcam ab7349; 1/1000), mouse anti-Smi32, monoclonal (Palex 23R-100; 1/2000), mouse anti-β (III) tubulin, monoclonal (Chemicon CBL412; 1/100), mouse anti-CD68, monoclonal (Serotec MCA341R; 1/200), mouse anti-NeuN, monoclonal (Millipore MAB377; 1/200), guinea pig anti-Iba1, polyclonal (Synaptic systems 234,004; 1/500), mouse anti-human mitochondria, monoclonal, fluorescence-labeled with Cy3 (Millipore MAB1273C3; 1/200). Secondary antibodies were labeled with fluorescent dyes: Goat anti-guinea pig IgG, Alexa-488 (Invitrogen A11073; 1/500), goat anti-rabbit IgG, TRITC (Sigma T5268; 1/500), goat anti-mouse IgG, Alexa-594 (Invitrogen A11005; 1/500), goat anti-mouse IgG, Alexa-488 (Jackson 115–545,003; 1/500), and goat anti-rat IgG, Alexa-488 (1/500).

### Microscopy and image analysis

Sections stained with H&E were photographed with a stereology microscope (Olympus BX61) using 4x and 10x objectives. For quantitative evaluation we determined the maximal anterior-posterior extension of the tissue lesion and the tissue loss in the lesion center of each rat. The latter was calculated by comparing the remaining tissue area in transverse spinal cord sections to corresponding sections of a rat without SCI.

Immunohistochemical staining was evaluated using a Leica epifluorescence microscope. Following visual inspection, objectives and exposure times were selected to account for different signal intensities obtained with different antibodies. Exposure conditions were held constant for quantitative evaluation with GFAP (5x objective), CD68 (10x), Iba1, Smi32, MBP (20x) and activated caspase-3 (40x). Photographs were analyzed using Fuji Image-J, applying the same brightness/contrast adjustments and threshold values for each marker.

The intensity of GFAP-immunoreactivity (IR) was measured as *integrated density* in regions of interest (ROI) in the glial scar around the lesion center; Iba-1: in the white matter in sections anterior and posterior of the lesion and in the lesion center; Smi32 and CD68: in the dorsal columns and in the ventrolateral white matter in sections anterior and posterior of the lesion. Signal intensities were normalized to values found in spinal cord sections from non-injured rats. For evaluation of apoptosis we counted cell nuclei that were IR for activated caspase-3 and expressed data as percentage of all nuclei in the ROI, which were located in dorsal and ventral grey matter, anterior and posterior of the lesion center (supplementary Fig. S1).

### Statistical analysis

Unless stated otherwise in the figure legends, data are presented as mean values +/− standard error of the mean (SEM). Non-parametric data are represented in a box and whiskers graph. The statistical analysis of weight and behavioral data (changes in time, effect of treatment), performed with GraphPad Prism v5 software, consisted of two-factor ANOVA, followed by post-hoc Tukey tests. For histological data, the differences among means were analyzed with unpaired Student’s t-test, considering *p* < 0.05 as statistically significant.

## Results

### Effect of bmSC implantation on the general health status, body weight and autonomic functions

The general health condition of the animals was not compromised. No adverse effects such as sickness behavior or tissue reaction to the bmSC were observed. Unexpectedly, one (bmSC-treated) rat was found dead after 29 dpo and one (MP treated) after 50 dpo. Pathological inspection of these animals did not show any significant abnormality. In five cases, biting at hind limbs occurred, but no animal had to be sacrificed due to sickness behavior or urinary infection.

**Fig. 2:**
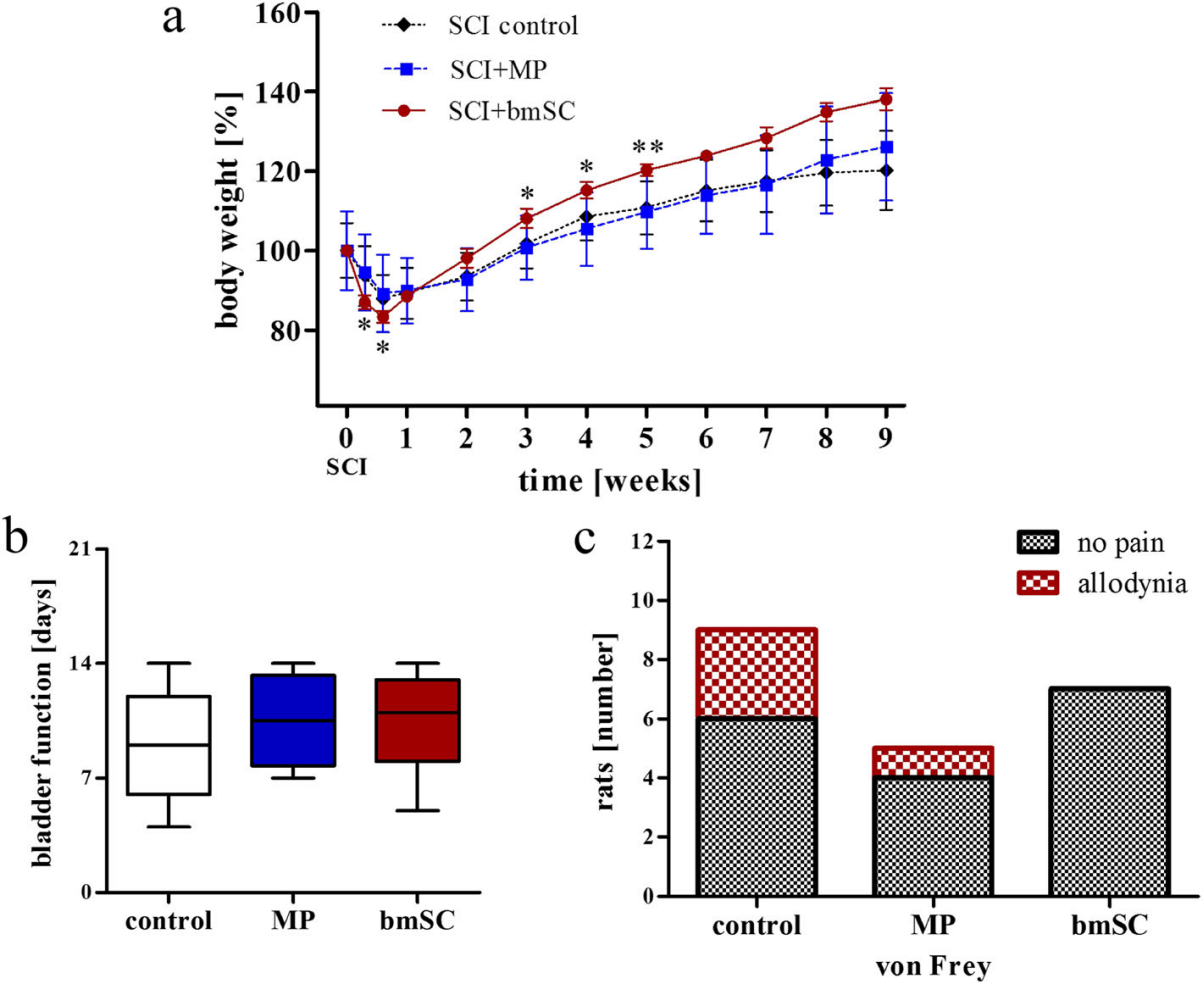
Health status after SCI. No adverse events were attributed to bmSC treatment. **a** Changes of body weight following SCI: Initially, stem cell-treated rats lost more body weight while at a later stage (starting at 14 dpo) they gained more weight compared to MP- and vehicle-treated animals. Data were normalized to the body weight before surgery (mean +/− SEM; two factor ANOVA, post hoc Tukey test, * *p* < 0.05, ** *p* < 0.01). **b** Recovery of the spontaneous micturition reflex: Displayed is the time after SCI [days] that passed until the animals no longer required manual voiding of the bladder by the experimenter (median, 25%/95% and range). There were no significant differences between treatment groups (H-test). **c** Testing of mechanical nociception (von Frey, reduced threshold of paw withdrawal response) at nine weeks after SCI showed no hyperalgesia/allodynia in bmSC implanted animals, while this occurred in 1/5 rats treated with MP and 3/9 rats that had received NaCl injections. Treatments following SCI are designated as: control - injections of 0.8% saline solution; MP - of methyl prednisolone; bmSC - of human bone marrow-derived stem cells

Following SCI surgery, body weight in all rats typically fell by 10–15% during the first 4 days and subsequently recovered with an average weight gain of about 8% per week during the first 5 weeks of the study and then slowing declining to 2% per week (Fig. 2a). Treatment was found to have a significant effect on relative change in body weight in bmSC-treated rats versus the control groups (interaction effect time x group, repeated measure ANOVA, F (7,56) = 8.83, *p* < 0.001). Post-hoc testing revealed that bmSC-treated rats initially lost significantly more weight than MP and vehicle-treated controls, but that they later on gained significantly more body weight compared to those groups (3 weeks after surgery: *p* = 0.042; 4 weeks: *p* = 0.018; 5 weeks: *p* < 0.01).

After SCI, animals needed assistance with bladder voiding, and all rats recovered autonomic bladder control within 2 weeks. Based on the volume of manually expelled urine, we found that the interventions with bmSC and MP did not significantly affect the return of spontaneous bladder control (Fig. 2b).

The response to tactile stimulation of the hind paws was tested manually with von Frey hairs before SCI surgery and after 9 weeks at the end of the study. Confirming the observations on overall health, none of the bmSC treated animals showed mechanical allodynia/hyperalgesia (lowered threshold of the paw withdrawal response). This was, however, the case in three animals of the NaCl control group and one rat of the MP treatment group (Fig. 2c).

### Tissue damage caused by the SCI

**Fig. 3:**
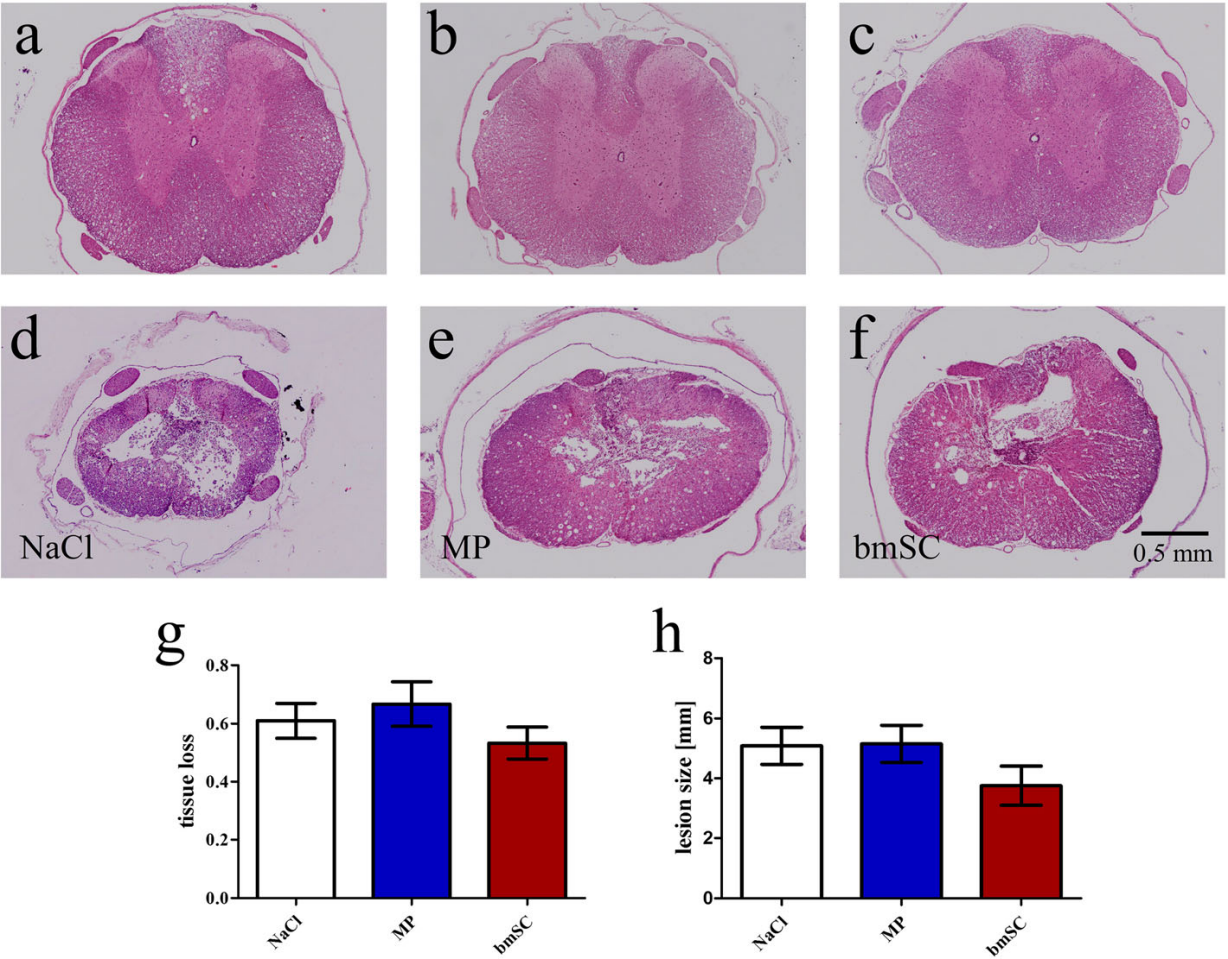
Treatment with bmSC and MP did not affect tissue degeneration. Lesion size and extent of tissue degeneration were evaluated in H&E-stained spinal cord sections at 10 weeks after SCI. **a-f** Panels show representative tissue sections 0.9 cm anterior of the lesion site (a-c) and at the lesion center (d-f); treatment groups were: **a, d** injection of saline; **b, e** methylprednisolone; and **c, f** human bmSC; same magnification in all photographs. **g** Relative tissue loss in the center of the lesion (normalized to spinal cord sections without lesion). **h** Anterior to posterior extension of lesion size as identified in H&E-stained spinal cord sections. Bars show means and SEM, *n* = 5–7 animals, differences between treatment groups were not significant

At the end of the study, the spinal cords of all animals were investigated with histology. Hematoxylin/eosin staining of transverse sections revealed extensive tissue damage caused by the contusion injury (Fig. 3). At the lesion center, more than half of the tissue was destroyed in all cases. Cavitation occurred, and to a large extent the remaining tissue contained non-neuronal scar, necrotic tissue and infiltration of inflammatory cells (Fig. 3a-f). Ependymal cells, identified morphologically, appeared to have proliferated. There were no quantitative differences between the treatment groups regarding the tissue loss in the center of the lesion (Fig. 3g). The anterior-posterior extension of the damage, which comprised all sections with pathological tissue alterations, was on average 4.7 mm (SD = 1.5 mm; Fig. 3h) without significant differences between treatment groups (t-tests, *p* > 0.05). The lesion centers, characterized by a fluid-filled cavity and scar formation, had an average size of 1.4 mm (SD = 1.1 mm) also without significant effect of treatment.

### Recovery of sensory-motor functions

At 2 dpo, 23 successfully operated animals scored < 2 in the BBB locomotor function scale (none or only slight movements of one or two joints; mean score of both hind legs), demonstrating a reasonable degree of reliability of the SCI rat model in our hands. Scoring differences between two blinded independent investigators were low (0–1), and in case of differences, the mean score of the two evaluators was recorded.

**Fig. 4:**
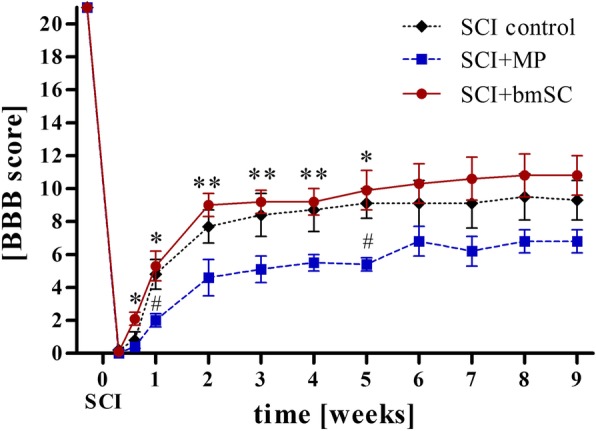
Injection of bmSC caused better recovery of motor function than MP treatment. Mean BBB scores (± SEM) of the three treatment groups. All rats had BBB = 21 before SCI, and the first evaluation occurred two days after surgery. As indicated in Fig. 1, only animals with BBB < 2 at 2 dpo (SCI considered as complete) were included in the evaluation. Following a two-factor ANOVA that revealed effects of treatment and time after SCI, post hoc Tukey test showed significant differences between bmSC and MP treatment (* *p* < 0.05, ** *p* < 0.01) and between NaCl and MP treatment (♯ *p* < 0.05)

Due to spontaneous recovery, time significantly affected motor function in all treatment groups (Fig. 4; *p* < 0.001), and a significant interaction effect was found between the groups and the treatment over time (repeated measure ANOVA, F (7, 56) = 5.75, *p* < 0.001). Importantly, rats treated with bmSC had significantly better motor function (BBB scores) compared to MP-treated rats at 4 days (*p* = 0.015), 7 days (*p* = 0.029), 2 weeks (*p* = 0.008), 3 weeks (*p* = 0.005), 4 weeks (*p* = 0.009) and 5 weeks (*p* = 0.015) after surgery (Fig. 4). After this time, mean score differences between bmSC and MP treatment remained in the same order of magnitude (ΔBBB was 4.5 at week 5 and 3.9 at week 9). These results indicate that the bmSC implantation resulted in better motor improvement than standard MP therapy. The effect was noted already at 4 dpo and reached highly significant levels during 5 weeks of recovery. Differences in recovery between bmSC and NaCl treatment, however, were smaller (ΔBBB was 0.8 at week 5 and 1.5 at week 9) and did not reach significance.

In addition to evaluation in the open field, rats were subjected to the Rotarod test. At 4 dpo none of the animals that met the inclusion criterion (BBB < 2) was able use their hindlimbs to maintain balance on the rotating bar. Spontaneous recovery caused a significant increase in Rotarod score during the first 4 weeks in all experimental groups (supplemantay Fig. S2). After 4 weeks, motor performance in this test did not improve further and in the saline treated animals actually deteriorated thereafter. Several rats refused the task as they did not grasp the bar with their fore paws. Their low score contributed to a very high variability. Thus, although bmSC treated animals had higher mean scores throughout the period of evaluation than both control groups, these differences were not significant (supplementary Fig. S2).

### Effect of bmSC and MP treatment on the astrocytic scar

**Fig. 5:**
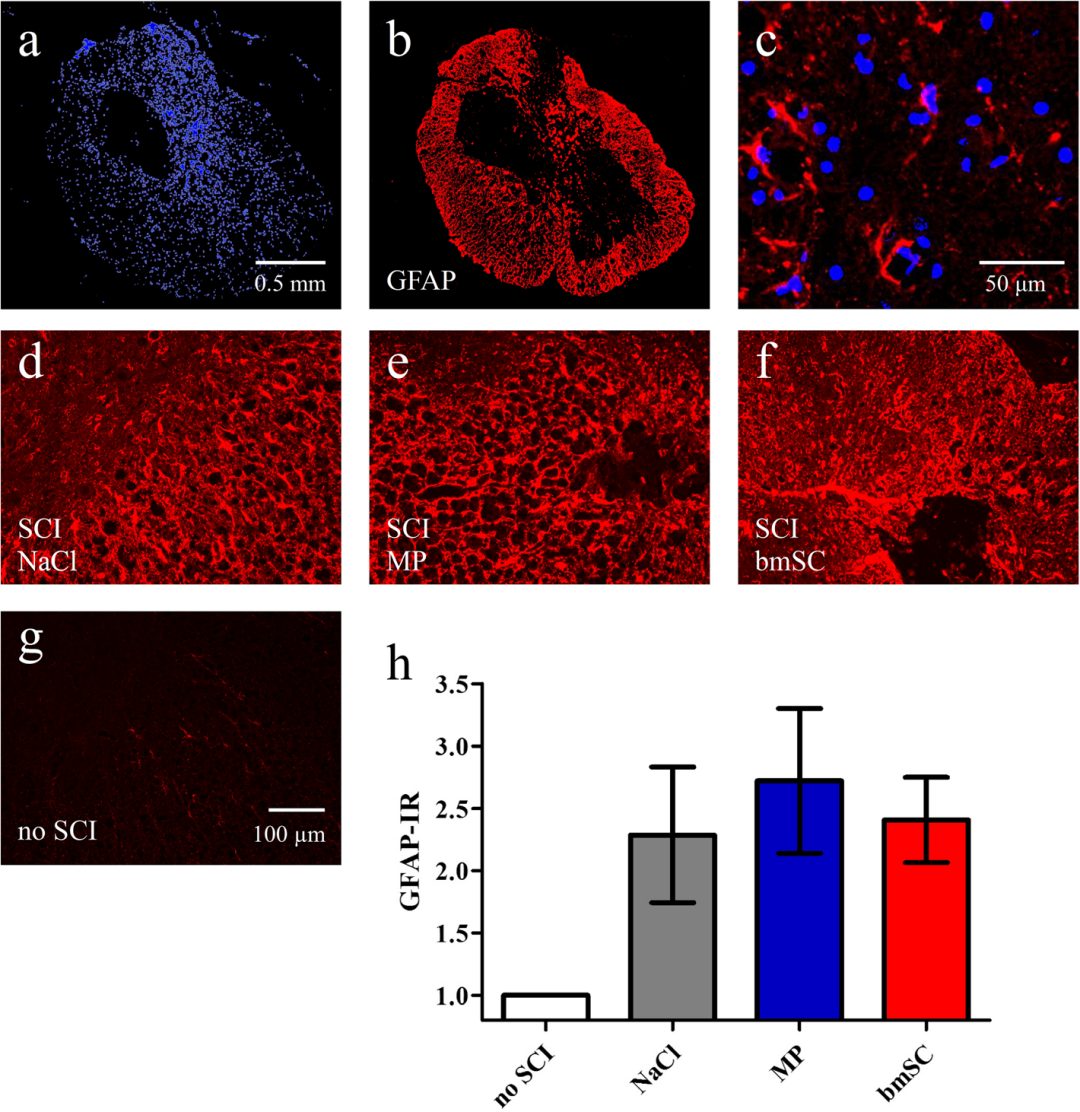
Astrogliosis was not affected by bmSC and MP treatment. Evaluation of GFAP-IR in spinal cord tissue ten weeks after SCI. **a-b** Overview of scar formation around the lesion center in a typical example; nuclear labeling with Hoechst-33342 (a) was combined with GFAP (b) immunostaining; 5x objective, scale bar 0.5 mm in a. **c** Reactive astrocytes in the gey matter outside of the lesion center. **d-f** Higher magnification of GFAP-IR close to the lesion site in SCI rats with control treatment (d), MP injections (e), bmSC implants (f), and **g** in the white matter of an animal without SCI; 20x objective, images d-g with the same times of exposure, scale bar 100 μm in g. **h** Quantification of GFAP-IR (integrated density) near the lesion site revealed no significant differences between SCI treatment groups (t-tests, *p* > 0.5). Data were normalized to GFAP-IR in the white matter of rats without lesion (statistical difference not indicated); bars show means and SEM, *n* = 5–6 rats/group

Astrocytes were visualized with GFAP-IHC in spinal cord sections containing the lesion site and in anterior and posterior sections without tissue alterations. This showed a dramatic increase of GFAP staining around the lesion center (Fig. 5a-g), indicating a persistent astrocytic scar in the chronic stage at 10 weeks after SCI. Treatment conditions had no significant effect on the GFAP-IR neither in white matter outside the lesion area nor in the center of SCI (Fig. 5h).

### Effect of bmSC and MP treatment on microglia and macrophages

**Fig. 6:**
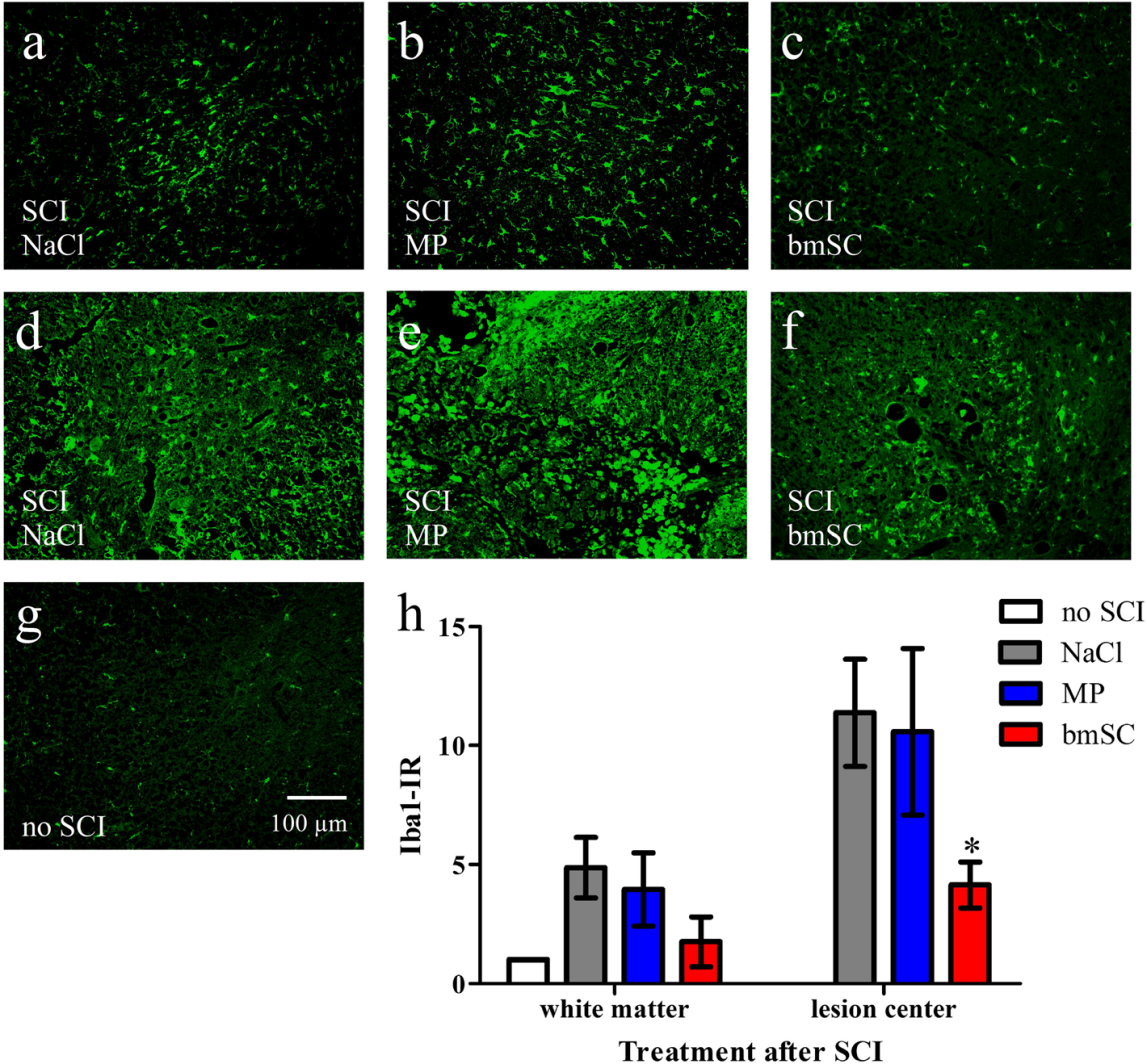
Injection of bmSC reduced activation of microglia/macrophages. Evaluation of Iba1-IR in spinal cord tissue ten weeks after SCI. **a-c** Microglia in spinal cord white matter 0.5–0.7 cm anterior of the lesion center. **d-f** Microglia and macrophages in sections containing the lesion center; representative examples from rats treated with saline (a, d), MP (b, e) and bmSC (c, f). **g** Microglia in the white matter of an animal without SCI; 20 objective, scale bar 100 μm valid for all photographs. **h** Quantification of Iba1-IR in the white matter ca. 0.8 cm anterior to and within the area close to the lesion center. Here, Iba1 expression was significantly lower after bmSC treatment compared to control treatment (t-test, * *p* < 0.5). Data were normalized to Iba1-IR in the white matter of rats without lesion (statistical difference not indicated); bars show means and SEM, *n* = 5–6 rats/group

Microglia and macrophages were stained with antibodies against Iba1, again using sections including the lesion site, anterior and posterior of this region (Fig. 6a-g). In the white matter of spinal cord sections outside of the area directly affected by the SCI we found cells with typical microglia morphology. Their Iba1 expression was 2- to 5-fold stronger compared to the white matter of rats without SCI. In the lesion center, Iba1-IR increased about 10-fold in animals treated with saline or MP but only 4-fold in animals that had received bmSC implants (Fig. 6h). Compared to the control treatment (NaCl injections) the effect of bmSC was significant (t-test, *p* < 0.05), indicating that the injected cells might have reduced activation of microglia or macrophages.

### Effect of bmSC and MP treatment on axon pathology

**Fig. 7:**
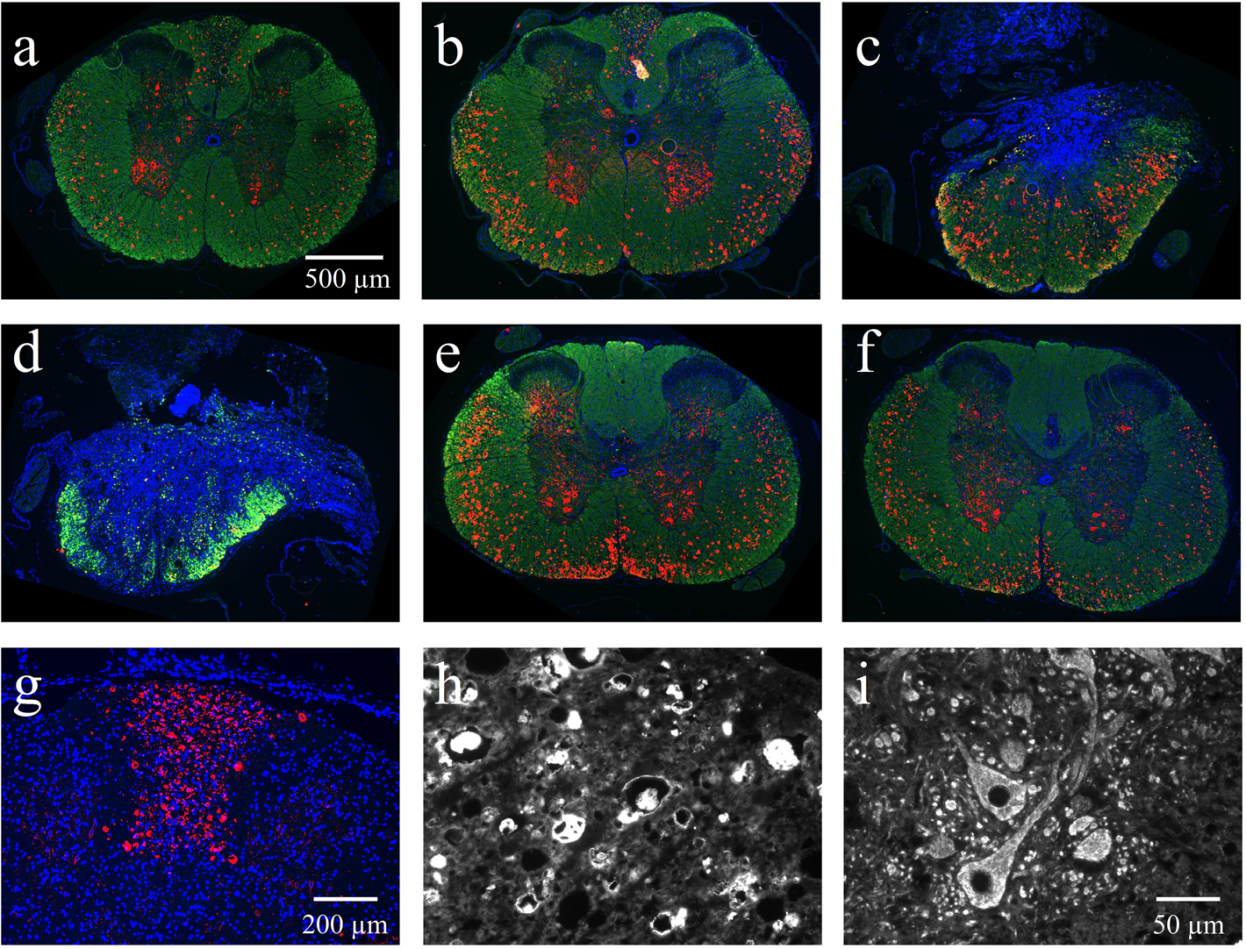
The presence of non-phosphorylated filaments as an indicator of axonal damage. Ten weeks after SCI immune staining with Smi32 antibody (red) was combined with myelin basic protein-IR (green) and Hoechst-33342 nuclear staining (blue). **a-f** Overview of transverse spinal cord sections at intervals of approximately 3.2 mm from 8 mm anterior to 8 mm posterior of the lesion site; 5x objective, scale bar in a. Note the presence of Smi32-binding in the ascending dorsal columns anterior but not posterior of the lesion site and in white matter tracts in all sections. **g** Non-phosphorylated neurofilament in ascending fiber tracts anterior of the site of injury, 20x objective. **h-i** Higher magnification of Smi32-IR in white matter (h) and motor neurons in the ventral horn (i), 40x objective, scale bar in i. No Smi32 staining was observed in the white matter of animals without SCI (see Fig. 8)

Non-phosphorylated neurofilaments are associated with their disassembled state in neuronal cell somata. In mature axons, in contrast, neurofilaments are heavily phosphorylated. Since this depends on myelin signals, the presence of non-phosphorylated neurofilaments in fiber tracts is indicative of demyelination and axonal damage [21, 22]. We investigated this using the monoclonal antibody Smi32, which labels non-phosphorylated neurofilament-M and -H [23]. Immune staining was observed in the white matter tracts of all SCI animals but not of non-injured rats. In the ascending dorsal columns the local Smi32-IR was particularly prominent in sections above the lesion site. In contrast, it was absent in the dorsal area of sections containing the lesion site, where all fiber tracts had completely degenerated, and also in the dorsal columns below the lesion, where these axons were not affected by the SCI (Fig. 7a-h). In ventrolateral fiber tracts, non-phosphorylated neurofilament was found in all spinal cord sections of lesioned rats. In the grey matter, Smi32-IR was also visible in the somata of nerve cells, most strongly in the ventral horns (Fig. 7i). This could also be observed in tissue of non-injured animals and is not pathological.

**Fig. 8:**
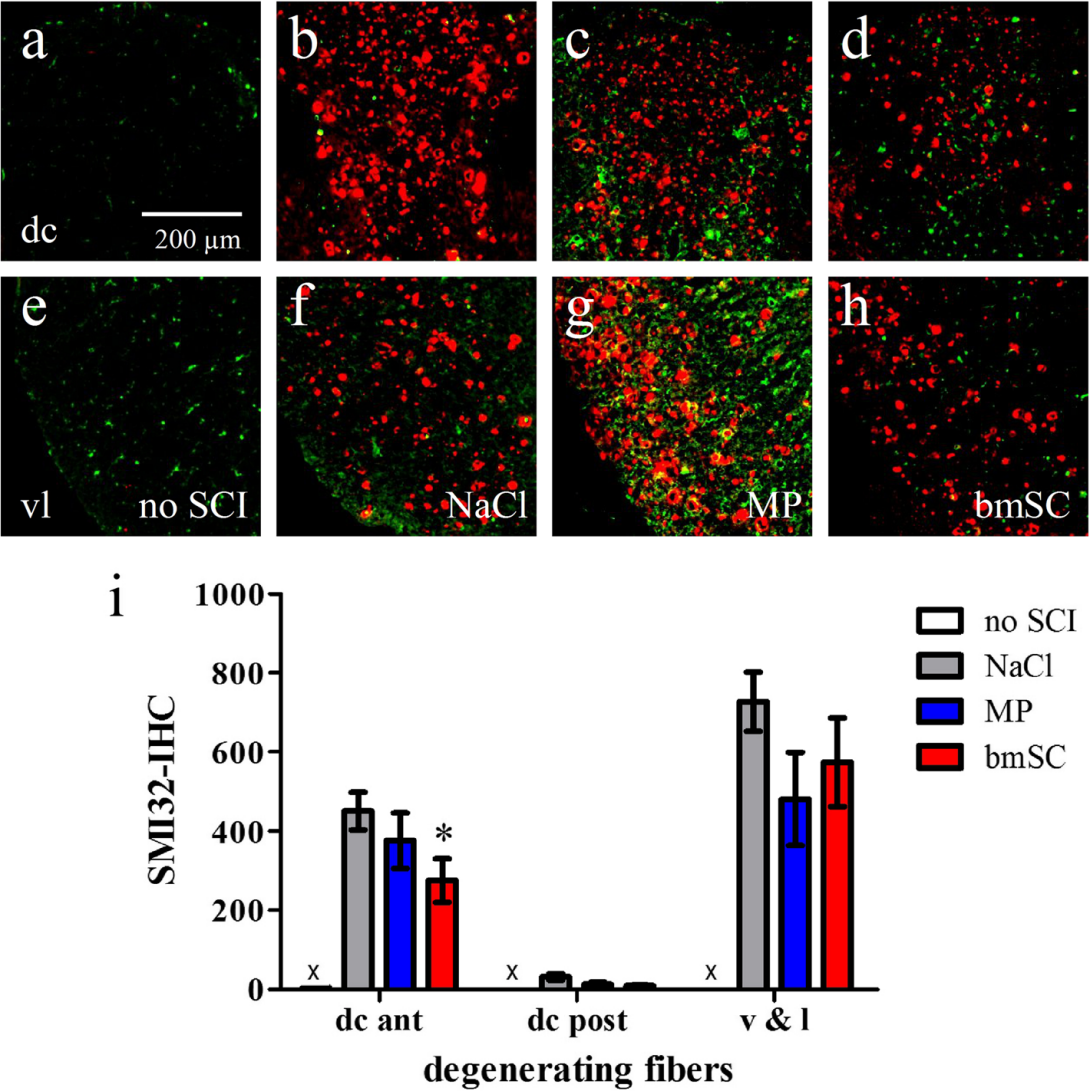
Treatment with bmSC reduced axonal damage in ascending fiber tracts anterior of the lesion site. Staining with Smi32 (red) was combined with Iba1 (green) in spinal cord tissue ten weeks after SCI. **a-h** Smi32 IR in the dorsal columns (a-d) and ventrolateral white matter (e-h) of a rat without SCI (a, e), and of SCI animals treated with saline (b, f), MP (c, g) and bmSC injections (d, h); 20 objective, scale bar in a. Note the absence of non-phosphorylated neurofilament in control samples without SCI in a and e. **i** Quantification of Smi32-IR in the ascending dorsal columns anterior and posterior of the lesion site (dc ant, dc post), the ventrolateral white matter (v&l) and corresponding regions without SCI (no Smi32-IR, marked x). Bars show means and SEM, *n* = 5–6 rats/group. Treatment with bmSC was associated with reduced Smi32-IR in the anterior dorsal columns compared to saline treatment (t-test * *p* < 0.05), while MP had no effect and differences in dc post and vl were not significant

Quantification of the Smi32-IR revealed a significant effect of bmSC treatment in the dorsal columns anterior of the lesion site, where the ascending somatosensory axons were affected by the SCI (Fig. 8a-d, i). Stem cell treatment reduced the amount of axonal damage compared to saline treatment (t-test, *p* < 0.05). The strong expression of non-phosphorylated neurofilaments in ventral and lateral fiber tracts was not significantly different between treatments (Fig. 8e-h, i).

### Effect of bmSC and MP treatment on neuroinflammation

**Fig. 9:**
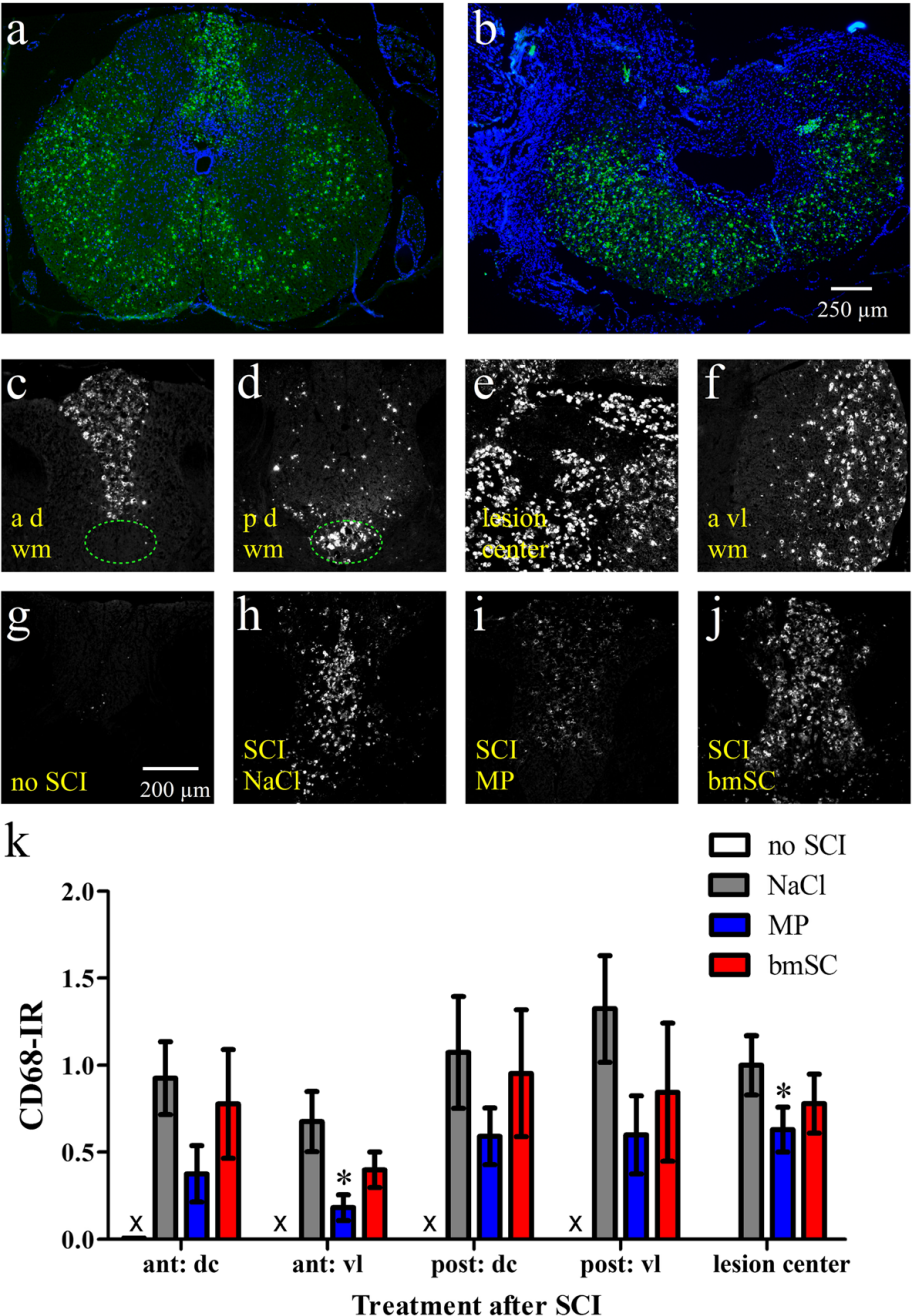
Injections of MP mitigated SCI-induced neuroinflammation. Microglia activation and macrophage infiltration ten weeks after SCI were evaluated with immune staining of CD68 (ED1). **a-b** Overview of transverse spinal cord sections 0.8 cm anterior of the lesion site and at its center. CD68 (green) was combined with Hoechst-33342 nuclear staining (blue), 5x objective, scale bar in b. Note very strong CD68-IR everywhere in the white matter as well as its absence in the scar tissue (b). **c-f** Examples of activated microglia/macrophages in ascending fiber tracts in the dorsal columns anterior of the lesion site (c), in corticospinal tract posterior of the lesion center (d; marked with dotted ellipse in c and d), in the lesion center (e), and anterior ventrolateral white matter (f). **g-j** Examples of CD68-IR in dorsal columns of rats without SCI and after SCI treatments; 10x objective, scale bar in g. **k** Quantification of CD68-IR in the dorsal columns (dc) and ventrolateral white matter (vl) anterior and posterior of the lesion site and corresponding regions without SCI (no CD68-IR). Bars show means and SEM, *n* = 5 rats/group. As indicated (t-test * *p* < 0.05) treatment with MP was associated with reduced CD68-IR compared to saline treatment. Injections of bmSC had no significant effect

To a large degree the devastating effects of SCI are due to a persistent neuroinflammatory response, one of its hallmarks being the lysosomal antigen CD68 (ED1), which is present in activated microglia and macrophages [24]. We found that activation of these myeloid cells was still very strong at 10 weeks after SCI (Fig. 9a, b). The histological distribution of CD68 IR throughout the white matter resembled that of axonal damage. Chronic neuroinflammation was observed in fiber tracts distal of the lesion, such as ascending somatosensory fibers anterior and the descending corticocpinal tract posterior of T9/T10 (Fig. 9c, d). Activated microglia and macrophages were also present in the lesion center and in ventral and lateral white matter tracts (Fig. 9e, f). Quantification of CD68-IR revealed that it was lower in MP treated animals than after NaCl treatment, while no significant effects were found after bmSC injection (Fig. 9g-k).

### Effect of bmSC and MP treatment on apoptosis

**Fig. 10:**
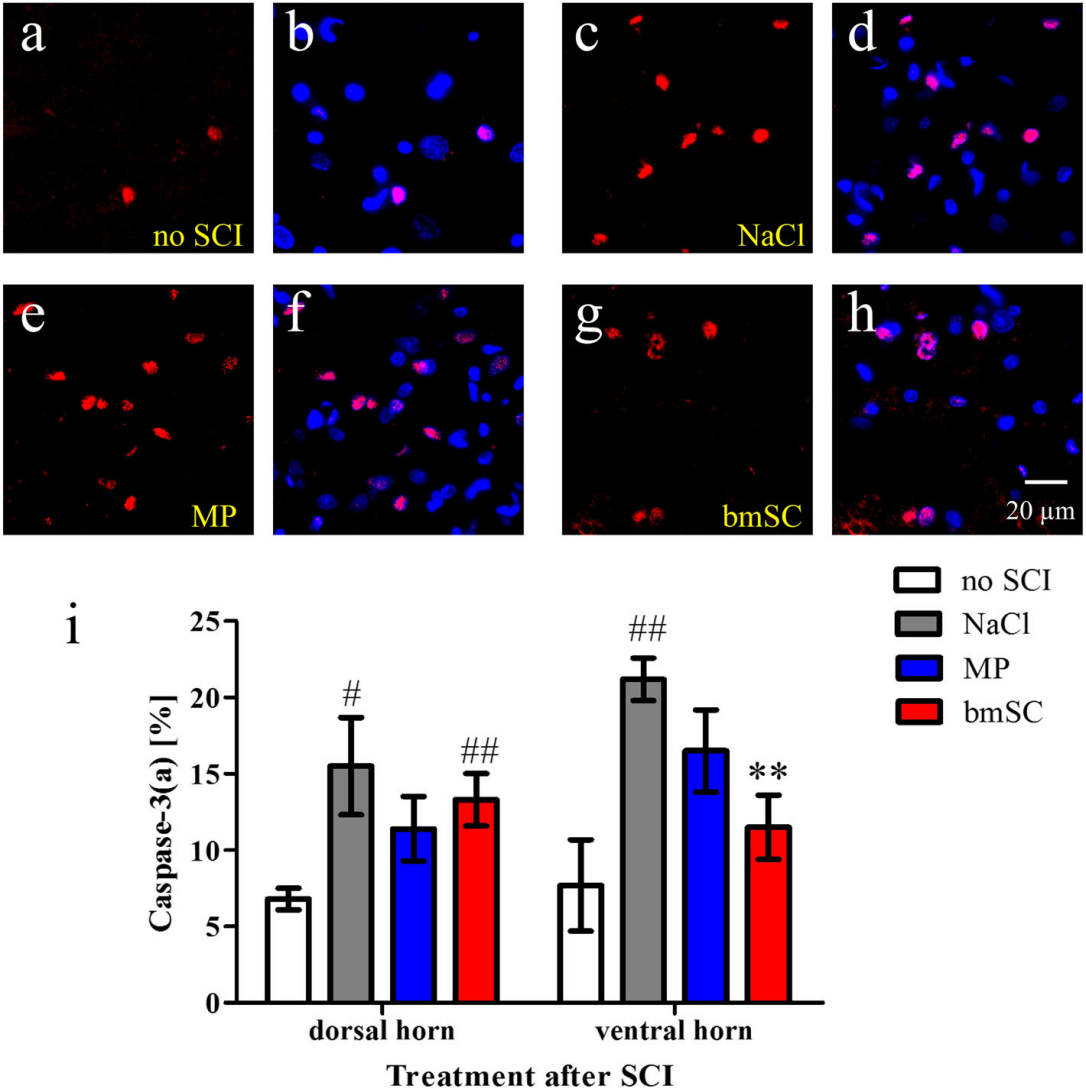
Injection of bmSC reduced apoptosis in the ventral horn. Ten weeks after SCI, cellular apoptosis was evaluated using activated caspase-3 as a marker. **a-h** Representative ROI containing apoptotic nuclei in the ventral horns of non-injured animals (a, b), after SCI/treatment with saline (c, d), with MP (e, f) and with bmSC (g, h). Immune staining of activated caspase-3 (red, all panels) was combined with Hoechst-33342 nuclear staining (blue, b, d, f, h, double exposure); 40x objective, scale bar in h. **i** Quantification of apoptosis in the grey matter is expressed as the percentage of activated caspase-3 IR nuclei of all nuclei. Bars show means and SEM, *n* = 5 rats/group; statistical evaluation with ANOVA, post-hoc Tukey tests. More apoptosis was observed after SCI when rats were treated with saline (♯ *p* < 0.05, ♯♯ *p* < 0.01). This increase in number of apototic cells failed to be significant after MP treatment and in the ventral horn also after bmSC treatment. Compared to saline, bmSC injections caused a highly significant reduction of apoptosis in the ventral grey matter (** *p* < 0.01)

Apoptosis was evaluated using an antibody against activated caspase-3, which at 10 weeks after SCI was clearly identified in cell nuclei (supplementary Fig. S1, Fig. 10a-h). While some apoptotic cells were also detected in the grey matter of rats without SCI (7% of all nuclei), the percentage was much higher (15–20%) in the rats with spinal cord contusion. Treatment with bmSC significantly reduced apoptosis in the ventral horn (Fig. 10i).

## Discussion

The therapeutic benefit of human bmSC as a treatment of SCI was compared to high dose MP injections in adult rats. Within 2 h after T9/T10 spinal cord contusion one dose of a dedicated human bmSC preparation (Neuroplast BV) was injected into the cisterna magna. These allogeneic implants were not rejected and significantly improved the recovery of motor functions compared to MP treatment. The mean BBB score of bmSC treated rats after 9 weeks was 10.8 compared to 6.8 after MP intervention. Differences between bmSC and saline injections (score of 9.3) were smaller and did not reach significance (Figs. 2, 4). On the histological level (Figs. 3, 5-10), bmSC treatment was beneficial with respect to axonal degeneration and apoptosis, compared to both control groups, while MP only had an anti-inflammatory effect.

To date, more than 25 studies have been published using bone marrow-derived stem cells to treat SCI in rats [8, 15, 25, 26]. In the past, the cells were usually expanded before use and their phenotype was not characterized. For the present study, we prepared human bmSC solely by removing immune cells from the bone marrow extract and did not cultivate them before implantation. Based on characterization with flow cytometry the injected cell suspension contained about 8% stem cells with a roughly equal proportion of hematopoietic and mesenchymal cells. How effective was this treatment in comparison with previous approaches? Even with standardized methods of evaluating motor function (BBB, rotarod) it is difficult to compare the outcomes reported by different laboratories (cf. BBB-scores of SCI control groups in [13, 25, 27]). With this caveat we may conclude that the therapeutic benefit of the new human bmSC preparation in rats was similar to what has been achieved using autologous cells. In the rat SCI model of severe contusion injury, no stem cell treatment has so far succeeded in repairing the tissue loss in the lesion center. Despite this, a benefit on functional recovery is observed justifying clinical trials [8, 28].

### Steroid treatment and limitations of the present study

Many publications on SCI treatment with MP report small to moderate improvement of motor recovery in the first weeks compared to placebo treatment. These are attributed to a reduction of inflammation, oxidative stress and neuronal apoptosis [29]. However, an absence of therapeutic benefit or even negative effects were also found [30]. In monkeys, MP inhibited the SCI-induced proliferation of ependymal stem cells in the spinal cord [31]. A meta-analysis of animal experiments concluded that “*beneficial effects of MP administration were obtained in 34% of the studies, no effects in 58%, and mixed results in 8%. The results were inconsistent both among and within species, even when attempts were made to detect any patterns in the results through subgroup analyses*” [32]. Due to its privileged role as the only FDA-approved pharmacological intervention in human SCI patients, MP is nonetheless often included in pre-clinical research. Following consultation with the EMA, we treated our rats with five intraperitoneal injections of 30 mg/kg MP with the first dose immediately after surgery and the following over 24 h, similar to the NASCIS II trial [4]. Compared to saline injections, this treatment significantly attenuated inflammation as shown with CD68 staining ten weeks after SCI (Fig. 9). Unexpectedly, it reduced motor recovery of the rats (Fig. 4).

Ethical principles in animal experimentation demand the largest possible reduction in the number of animals. Based on expected effect size and variance we planned eight rats for the treatment and six for the three different control groups. While a highly significant benefit of bmSC compared to MP treatment was reached (Fig. 4) and differences with all control groups were significant on the histological level (Figs. 6-10), this design was underpowered to demonstrate a functional benefit of bmSC compared to saline treatment. Additional tests with the Rotarod assay indicated a positive influence of bmSC on motor recovery compared to both control groups, however these data did not reach significance because of their high variability (supplementary Fig. S2). This was primarily caused by the fact that 1/2 to 1/3 of the rats, irrespective of treatment, did not try to hold on to the rotating bar, although all animals had successfully been trained to do the task prior to SCI. Increasing body weight of the animals appeared to make the task more difficult during the study.

In a future clinical application the bmSC are intended to be extracted from the same person who suffered the SCI and will receive the treatment. The time between bmSC preparation and injection shall not exceed 48 h (Neuroplast, patent WO2015/059300A1). Deviating from this procedure we tested the human cells in rats. Since it was not possible to implant the cells immediately after their preparation, bmSC were cryopreserved and resuspended for implantation, and this reduced their viability. Of all nucleated cells in the bmSC preparation 3.3% were hematopoietic stem cells (CD34), 3.8% mesenchymal stem cells (CD271, CD90, CD105, CD73) and the rest were non-identified stroma cells also including dead cells [cf. 13, 33]. Despite these limitations, the implants were not rejected, the treated animals showed no sickness behavior and a better recovery of body weight than control groups (Fig. 2). We attribute this success to the properties of the human bmSC as modulators of innate immunity.

### The advantages of bmSC implants as a therapy of SCI

Today, stem cell based therapies are among the most promising experimental strategies to treat neurodegenerative pathologies including SCI. As an advantage compared to other sources, such as embryonic and induced pluripotent stem cells (iPCS), adult stem cells are easily isolated from blood, bone marrow or adipose tissue. In contrast to iPSC [16, 33] they do not require genetic reprogramming and pose no risk of tumor formation. Several attempts using bmSC in rodents have achieved significant improvements in motor functions, which were in the same order of magnitude as in the present study [7, 8]. Despite the inherent difficulties to publish negative results, some failures to reach functional improvement have also come to light [7, 34, 35], and this raises the question as to the best conditions for bmSC treatment of SCI. Three considerations deserve particular attention: preparation of the bmSC, mode and time of application.

1) Following standard extraction of bone marrow from the iliac crest of human donors, we are using a novel procedure to prepare bmSC, which is based exclusively on the elimination of macrophages and lymphocytes without manipulation or expansion in vitro. This procedure allows implantation within 48 h after harvesting of the cells. In most of the previous studies bmSC were expanded to large numbers before use [7, 8] and this, unfortunately, reduces their growth potential (Hayflick limit [36]) as well as their anti-inflammatory properties [37]. It also leads to the accumulation of stochastic mutations, such that the risk of malignant transformation cannot be ruled out [38]. A major advantage of our approach is to avoid negative changes associated with long term cultivation.

2) Cell implants that are intended for the therapy of CNS pathologies first need to reach their target tissues. While contusion SCI initially disrupts the blood-spinal cord barrier, this is restored by endogenous repair processes. Therefore, systemic applications of cells, such as by intravenous injection [39, 40], may have only a limited time window, which in rodents lasts about one week for the gray matter [3]. Since we intend to explore treatment in the chronic phase in the future, we chose infusion into the cerebrospinal fluid (CSF). Stem cell injections into the CSF were shown to be more effective than into the blood circulation [40–42], and in the majority of clinical studies cells were transplanted via lumbar puncture [8, 43]. In rats, we accessed the subarachnoid space via the cisterna magna [42], implying that the injected cells have to migrate toward the area of injury in the spinal cord. While the mechanisms of this are not well understood, homing to damaged areas has been shown to be a property of bmSC even when injected into the blood stream [39]. We considered the alternative to inject the cells directly below the dura mater of the spinal cord, as was done previously after dorsal column transection [44] and compression injury [13]. However, in preparatory experiments we found that spinal cord injection per se caused additional damage. This application may be more effective for interventions in the chronic phase, when cells can be implanted into the cavity within the spinal cord that has formed by then [11, 28].

3) Thus, the time of intervention is another crucial parameter when considering stem cell therapy of SCI. By far the most animal experiments have been carried out in the acute phase, and with bmSC this seems to be justified because their main benefit is expected to be neuroprotection by modulating the immediate inflammatory response [3, 13, 45]. Our histological evaluation indicates that the acute intervention, while not reducing gross tissue damage (Fig. 3), did have lasting cytoprotective effects as shown with a reduction in axonal damage (Fig. 8) and apoptosis (Fig. 10) ten weeks later. Since immune suppression of macrophages was larger after MP treatment (Fig. 9), we hypothesize that the bmSC elicited additional neurotrophic effects. These will be explored in future SCI experiments using intervention in the chronic state. In a delayed treatment protocol with intraspinal administration into the lesion cavity the integration of grafted cells promises to be better because the release of toxic compounds, lytic enzymes and free radicals of the early phase has somewhat subsided [8].

### The putative mode of action of bmSC after SCI

This raises the question regarding the mechanisms by which the injected bmSC were effective in our experiments. Increasing evidence suggests that extracellular vehicles (EVs) are important players in mediating the therapeutic effects of therapeutically applied stem cells [15, 26, 46, 47]. Exosomes from mesenchymal stem cells exert immune-suppressive effects by enforcing M2 macrophage polarization, inhibiting complement activation [26] and indirectly driving regulatory T cell induction [14]. In addition, classical mechanisms of paracrine release of cytokines and growth factors are likely to be involved [48, 49], although attempts at isolating these factors so far have failed to replace stem cells with a pure pharmacological intervention. Stem cell-conditioned media which contain EVs as well as paracrine factors can be effective, although repeated delivery may be required [15, 46]. It is believed that beneficial effects of bmSC are derived rather from the mesenchymal and not the hematopoietic stem cell fraction [8], and this view is linked to the expectation that the cells integrate and differentiate in the tissue [49]. Our bmSC preparation contained less than 5% mesenchymal stem cells. We have reason to believe that hematopoietic stem cells and remaining stromal cells (not expressing CD34, CD271, CD90, CD105, CD73) also released modulators that positively influenced recovery after SCI. Although the injected bmSC reduced Iba1 staining, their effect on CD68 did not reach significance. The fact that cell treatment improved motor recovery much better than MP, while the latter did reduce the number of CD68 positive macrophages, also indicates that bmSC may have acted on other than myeloid cells.

The formation of fibrotic and glial scar is a major impediment to axonal regeneration after SCI. While reports with bmSC have claimed to reduce this [8, 13, 15], we did not see differences in scar formation or lesion size between different groups. Either there was a transient effect, not visible ten weeks after SCI, or the damage caused by a 200 Kdyn (2 N) contusion injury was simply too large to put any scar reducing effects in evidence.

Were there continuing effects in the chronic stage? The behavioral data show that almost all improvement in sensory-motor performance of the rats occurred within the first three weeks and that the therapeutic benefit of bmSC treatment also occurred in this period (Fig. 4). Using a specific antibody against human mitochondrial proteins (Millipore MAB1273C3, validated in vitro) we searched for the presence of human cells in the spinal cords of all rats. At ten weeks after SCI we were not able to detect the implants. Although the absence of an IR signal is certainly not conclusive, it is more likely that the implanted bmSC were only effective in the acute and subacute phase after SCI. Differences observed after ten weeks on the histological level, such as lower microglial activation and reduced axonal damage may be the result of better recovery in the subacute phase. This must certainly be the case for the lingering anti-inflammatory effect of acute MP injections. It is intriguing, though, that we observed a high level of apoptosis and a significant effect of bmSC on this phenomenon even at ten weeks after lesion. Double IHC with antibodies against activated caspase-3/Iba1 and activated caspase-3/NeuN indicated that the apoptotic nuclei did not belong to microglia or neurons (data not shown). Previous studies found continuing apoptosis of oligodendrocytes in the chronic phase after SCI [50]. Other groups [39, 44] were able to locate injected bmSC infiltrating the lesion site. In several cases, cells were found to have differentiated into oligodendrocytes, whereas the expression of neural markers was rare. Following the most thorough analysis of cellular transplantation therapies for SCI, Tetzlaff and colleagues [7] concluded that remyelination of demyelinated axons may be the most realistic therapeutic objective.

## Conclusions for improving SCI therapy based on bmSC implants

Using acute intervention with bmSC we were able to improve the natural recovery process within the first 10 weeks after lesion compared with corticosteroid treatment without adverse effects due to a possible immunological rejection. By reaching these objectives, the results of this study confirmed the beneficial effects of stem cells that were obtained earlier using immune-compromised rats and balloon compression SCI [13].

In this and many other studies implanted stem cells could not be identified in the tissue when this was attempted in the chronic stages after SCI. Their failure to survive may be attributed to a hostile microenvironment created by the lesion [12, 51]. It is therefore an objective to modify the tissue response such that implanted cells remain functional. Since the bmSC themselves modulate the innate immune system [26, 45, 47], we suggest that a combination of pharmacological/cell-based therapies should complement the signals released from the bmSC by activating different molecular targets.

In the past, even the most promising results of preclinical studies with rodents could not be translated to clinical therapies of SCI or any other neurodegenerative disease. Depending on the physiological question and the risk of treatment, additional studies with non-human primates may therefore be necessary before a clinical trial is justified [9]. Since large mammals are expensive, sample sizes are usually small. Such experiments are ethically justified only to the extent that the animal models are more predictive for clinical interventions than experiments with rodents. A recent comparative study with bmSC injections after SCI in 115 rats and 17 pigs arrived at similar results in both species [25]. The present results demonstrate that our bmSC preparation had benefits and no negative side effects even when implanted in a different species and with a considerable percentage of non-viable cells due to one freezing/thawing cycle before implantation.

In a clinical trial, bmSC would be prepared from the same patient and implanted without cryopreservation and within 48 h after injury. Since the potential risk for the patient is minimal we do not see the necessity of an intermediate study with large mammals or non-human primates, especially since differences in the motor systems between primate species are also not negligible [52]. Exaggerated promises are a recurrent phenomenon in SCI research. Our conclusion, while optimistic, is more modest: The intrathecal transplantation of human bone marrow-derived cells prepared via negative selection and without cultivation will contribute to a combinatorial therapy of SCI.

## Supplementary information

**Additional file 1 Fig. S1.** Evaluation of cellular apoptosis. Spinal cord sections were processed with double IHC against activated caspase-3/NeuN (neurons) and activated caspase-3/Iba1 (microglía/macrophages) and combined with DAPI nuclear staining. **a** Drawing of spinal cord and transverse sections indicating the ROIs for evaluation in the grey matter (40 x objective). **b** Low power photograph of the dorsal horn of a rat with SCI, demonstrating the distribution of apoptotic nuclei (pink). Annotation indicates outline of grey matter, ROI, counted cell nuclei and scale bar = 100 μm.

**Additional file 2 Fig. S2.** Motor recovery as revealed with the Rotarod test. Motor performance in the rotarod stepping test is expressed in the amount of time [sec] that the animals maintained themselves on the rotating bar (mean ± SEM); SCI + bmSC: treatment with human bone marrow-derived stem cells; SCI + MP: injections of methyl prednisolone; SCI control: injections of NaCl. Before SCI, all animals reached the maximum time of 300 s. The first evaluation was performed at 4 dpo. Differences between groups were not significant. Rats that did not attempt to hold on to the bar and therefore received a score of zero sec were not excluded from the statistical evaluation.

## Data Availability

The datasets used and/or analysed during the current study are available from the corresponding author on reasonable request.

## Abbreviations

BBB: Basso, Beatty, Bresnahan locomotor rating scale
bmSC: Bone marrow-derived stromal cells
CD: Cluster of differentiation
CSF: Cerebrospinal fluid
DMSO: Dimethyl sulfoxide
dpo: Days post operation
EDTA: Ethylenediamine tetraacetic acid
EMA: European Medicines Agency
FDA: U.S. Food and Drug Administration
GFAP: Glial fibrillary acidic protein
GMP: Good manufacturing practice
HSC: Hematopoietic stem cells
Iba1: Ionized calcium-binding adapter molecule 1
IH: Infinite Horizon spinal cord impactor
IHC: Immunohistochemistry
IR: Immunoreactivity
MBP: Myelin basic protein
MP: Methylprednisolone
NASCIS: National acute spinal cord injury study
PBS: Phosphate buffered saline
RT: Room temperature
SCI: Spinal cord injury
TBS-T: Trisaminomethane-buffered saline/0.05% Tween 20
TRITC: tetramethyl rhodamine iso-thiocyanate
