# Supplementary figures and images for: Treatment of rats with spinal cord injury using human bone marrow-derived stromal cells prepared by negative selection
Source: J Biomed Sci. 2020 Feb 18;27:35. doi: 10.1186/s12929-020-00629-y (PMC7026953; doi:10.1186/s12929-020-00629-y)

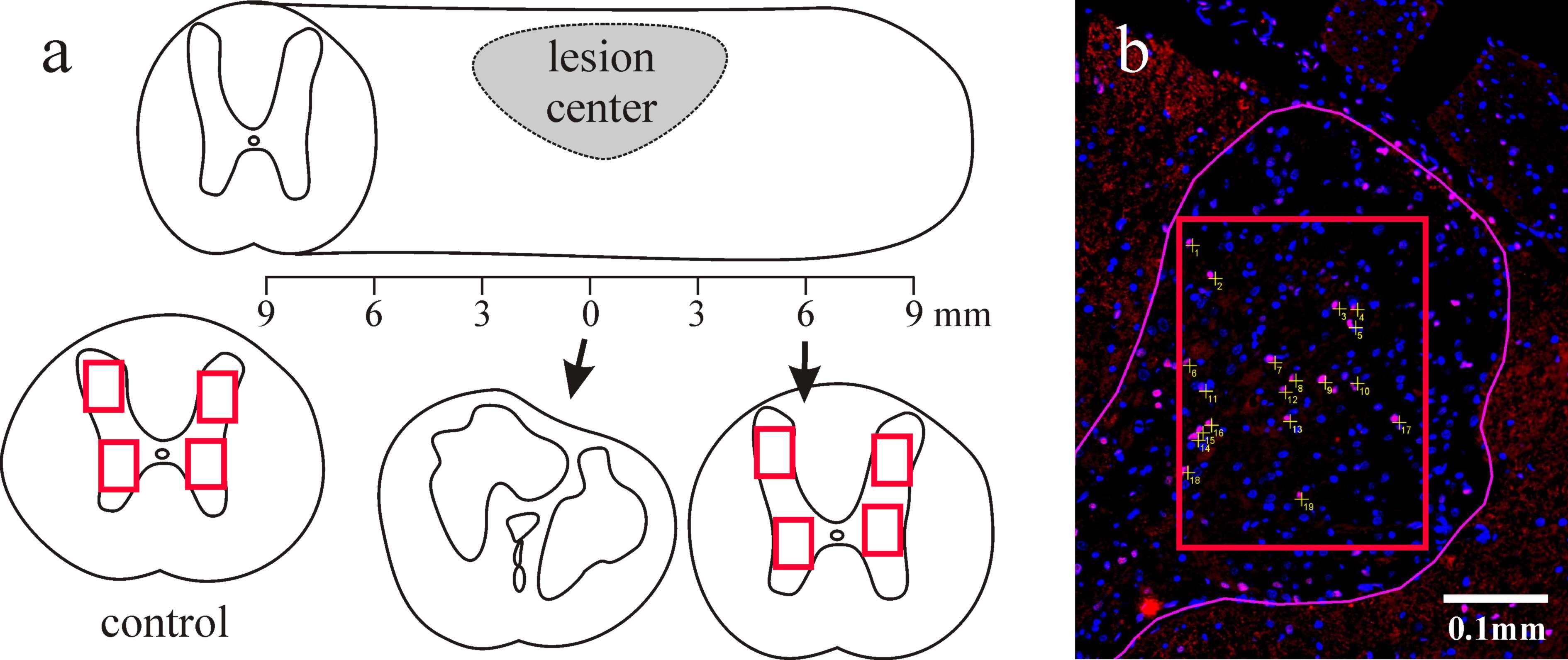

Supplement: Supplementary file 1 — Additional file 1 Fig. S1. Evaluation of cellular apoptosis. Spinal cord sections were processed with double IHC against activated caspase-3/NeuN (neurons) and activated caspase-3/Iba1 (microglía/macrophages) and combined with DAPI nuclear staining. a Drawing of spinal cord and transverse sections indicating the ROIs for evaluation in the grey matter (40 x objective). b Low power photograph of the dorsal horn of a rat with SCI, demonstrating the distribution of apoptotic nuclei (pink). Annotation indicates outline of grey matter, ROI, counted cell nuclei and scale bar = 100 μm. [file 12929_2020_629_MOESM1_ESM.jpg]

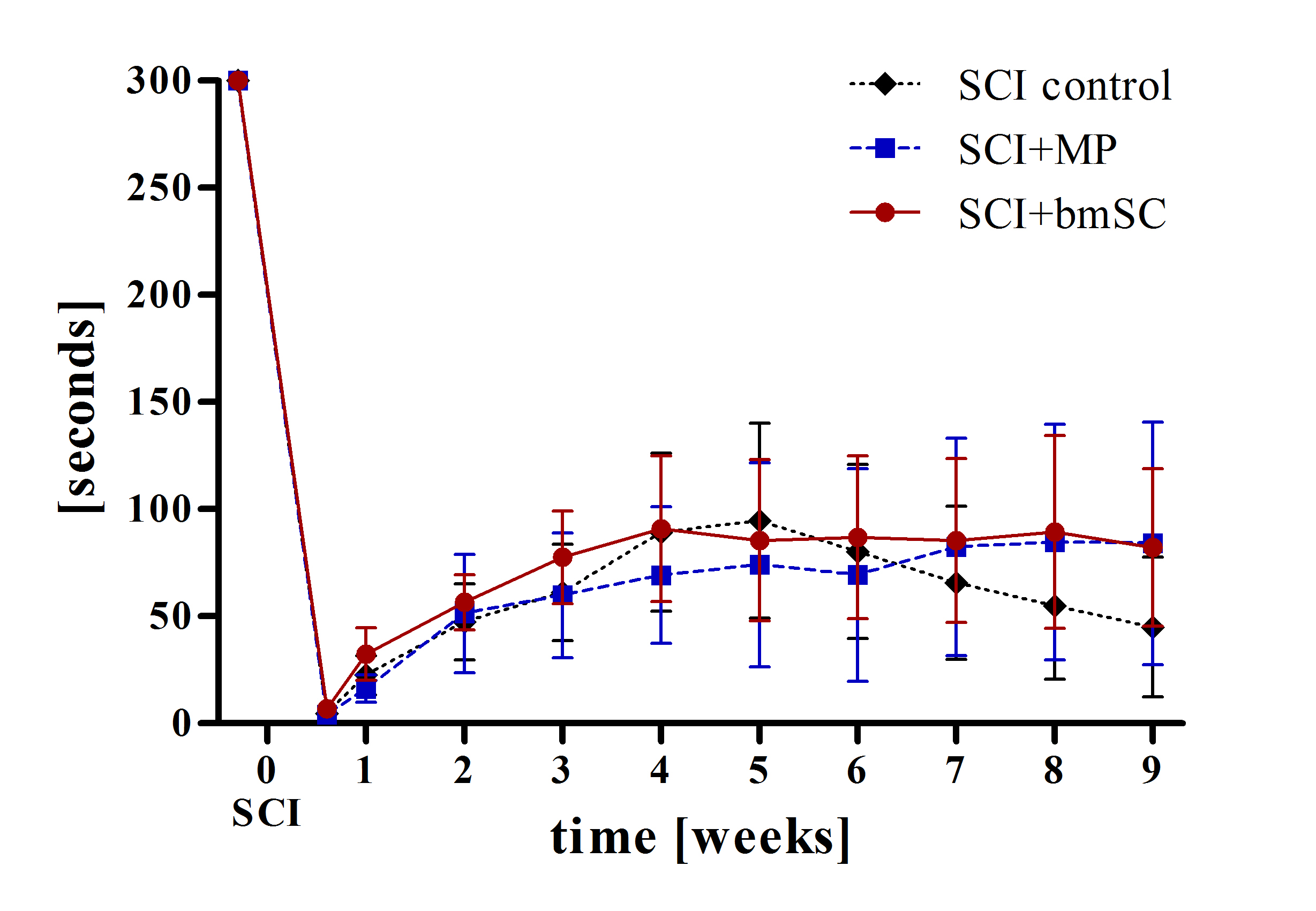

Supplement: Supplementary file 2 — Additional file 2 Fig. S2. Motor recovery as revealed with the Rotarod test. Motor performance in the rotarod stepping test is expressed in the amount of time [sec] that the animals maintained themselves on the rotating bar (mean ± SEM); SCI + bmSC: treatment with human bone marrow-derived stem cells; SCI + MP: injections of methyl prednisolone; SCI control: injections of NaCl. Before SCI, all animals reached the maximum time of 300 s. The first evaluation was performed at 4 dpo. Differences between groups were not significant. Rats that did not attempt to hold on to the bar and therefore received a score of zero sec were not excluded from the statistical evaluation. [file 12929_2020_629_MOESM2_ESM.jpg]
